# Proteomic-Based Machine Learning Analysis Reveals PYGB as a Novel Immunohistochemical Biomarker to Distinguish Inverted Urothelial Papilloma From Low-Grade Papillary Urothelial Carcinoma With Inverted Growth

**DOI:** 10.3389/fonc.2022.841398

**Published:** 2022-03-24

**Authors:** Minsun Jung, Cheol Lee, Dohyun Han, Kwangsoo Kim, Sunah Yang, Ilias P. Nikas, Kyung Chul Moon, Hyeyoon Kim, Min Ji Song, Bohyun Kim, Hyebin Lee, Han Suk Ryu

**Affiliations:** ^1^ Department of Pathology, Seoul National University College of Medicine, Seoul, South Korea; ^2^ Department of Pathology, Severance Hospital, Yonsei University College of Medicine, Seoul, South Korea; ^3^ Department of Pathology, Seoul National University Hospital, Seoul, South Korea; ^4^ Transdisciplinary Department of Medicine and Advanced Technology, Seoul National University Hospital, Seoul, South Korea; ^5^ Proteomics Core Facility, Biomedical Research Institute, Seoul National University Hospital, Seoul, South Korea; ^6^ School of Medicine, European University Cyprus, Nicosia, Cyprus; ^7^ Kidney Research Institute, Medical Research Center, Seoul National University College of Medicine, Seoul, South Korea; ^8^ Center for Medical Innovation, Biomedical Research Institute, Seoul National University Hospital, Seoul, South Korea; ^9^ Department of Pathology, Konkuk University Medical Center, Konkuk University School of Medicine, Seoul, South Korea; ^10^ Department of Radiation Oncology, Kangbuk Samsung Hospital, Sungkyunkwan University School of Medicine, Seoul, South Korea

**Keywords:** inverted urothelial papilloma, papillary urothelial carcinoma, transitional cell carcinoma (TCC), tandem mass spectrometry (MS/MS), machine learning analysis, immunohistochemistry, biomarkers, differential diagnosis

## Abstract

**Background:**

The molecular biology of inverted urothelial papilloma (IUP) as a precursor disease of urothelial carcinoma is poorly understood. Furthermore, the overlapping histology between IUP and papillary urothelial carcinoma (PUC) with inverted growth is a diagnostic pitfall leading to frequent misdiagnoses.

**Methods:**

To identify the oncologic significance of IUP and discover a novel biomarker for its diagnosis, we employed mass spectrometry-based proteomic analysis of IUP, PUC, and normal urothelium (NU). Machine learning analysis shortlisted candidate proteins, while subsequent immunohistochemical validation was performed in an independent sample cohort.

**Results:**

From the overall proteomic landscape, we found divergent ‘NU-like’ (low-risk) and ‘PUC-like’ (high-risk) signatures in IUP. The latter were characterized by altered metabolism, biosynthesis, and cell–cell interaction functions, indicating oncologic significance. Further machine learning-based analysis revealed SERPINH1, PKP2, and PYGB as potential diagnostic biomarkers discriminating IUP from PUC. The immunohistochemical validation confirmed PYGB as a specific biomarker to distinguish between IUP and PUC with inverted growth.

**Conclusion:**

In conclusion, we suggest PYGB as a promising immunohistochemical marker for IUP diagnosis in routine practice.

## Introduction

Inverted urothelial papilloma (IUP) is an uncommon neoplasm that accounts for less than 1% of bladder tumors (1). Although IUP generally exhibits a benign behavior, it has often been reported to show a synchronous/metachronous occurrence with papillary urothelial carcinoma (PUC), raising a concern of having an indeterminate malignant potential (1–3). The malignant potential of IUP has been reinforced by recent genomic data, where a high-risk subset of IUP was shown to harbor key driver oncogenes predisposing to PUC at the genomic level, including *FGFR3* and *TERT* promoter mutations, although the frequency varied between studies (4–6). From the perspective of diagnostic accessibility, nevertheless, it remains controversial whether genomic tests should always be used to predict the oncogenic risk of IUP. Notably, previous studies also have exhibited considerable discordance between genomic mutations and their corresponding protein levels (7, 8). Therefore, assessing the oncogenic potential at the protein level with immunohistochemistry would be preferred, as the latter is a technique widely practiced in diagnostic pathology.

Along with the difficulty to identify the high-risk IUPs, the absence of reliable biomarkers to differentiate IUP from low-grade PUC with inverted growth is another unsolved issue in pathology diagnostics. IUP and PUC with inverted growth are well-known to share several microscopic features, such as slender trabeculae and mild cytomorphological atypia, which could lead to misinterpretation (3). Some ancillary tests, namely, Ki-67, p53, cytokeratin 20, and HER2 immunohistochemistry and genomics assays such as *in situ* hybridization or next-generation sequencing might be helpful in this context (6, 9–12). However, the use of these approaches is hampered by their limited accuracy and applicability (10).

Recent advances in proteomics have enabled in-depth functional analyses of several types of tumors (13–15). In contrast to genomic analysis, the proteomic layer directly reflects proteins, the final units controlling cellular functions. In addition, proteomics-based analysis is more likely to successfully discover a protein-based biomarker that can subsequently be used in immunohistochemistry, the most widely used ancillary diagnostic tool in practice. In previous studies, we presented proteome-based novel diagnostic markers of bladder urothelial carcinoma in liquid-based cytology and Formalin-fixed paraffin embedded (FFPE) specimens (16, 17). In this study, we performed a clinical proteomic analysis to identify proteome-based molecular profiles stratifying the risk in IUP and discover a reliable protein biomarker for the pathological diagnosis of IUP.

## Materials and Methods

### Patient Selection and Clinicopathological Review

FFPE tissue specimens were procured from the Pathology Department of the Seoul National University Hospital. The diagnoses were reviewed by three board-certified pathologists (MJ, KM, and HR), using hematoxylin and eosin slides, according to the 2016 World Health Organization Classification (18). The IUPs included in this study showed inverted trabeculae, cords, or nests of thin urothelium with intact maturation pattern and no cytological atypia (**Supplementary Figure S1**). Any patient with a previous history of bladder tumor and/or intravesical treatment was excluded. Clinical information was obtained from the medical records. The regional Institutional Review Board (IRB) approved the experimental protocols (IRB No. H-2009-163-1160).

For proteomic analysis, 31 tissue specimens, consisting of 9 IUP, 12 PUC, and 10 normal urothelium (NU), were included (**Supplementary Table S1**). All IUP and PUC specimens were cystoscopically resected from the urinary bladder. All PUCs were non-invasive (stage Ta) and 83.3% (10/12) were high-grade. For validation, we performed immunohistochemical staining in an independent validation cohort composed of 25 IUP and 16 PUC with inverted growth (**Supplementary Table S1**). The inverted growth pattern accounted for variable portions (mean ± S.D., 52 ± 32%) of PUCs with inverted growth. The overall demographics of the specimens for validation were similar to those of the proteomic cohort, except most (81.2%) PUCs with inverted growth in the validation cohort were low-grade. All patients with IUP, except for one, were followed up for 12–52 months (median, 31 months) by urine cytology, cystoscopy, or computed tomography, and no one showed recurrence.

### Liquid Chromatography With Tandem Mass Spectrometry Analysis and Data Processing

Tandem mass spectrometry (LC–MS/MS) analysis was conducted following the methods used in our previous study (17). Briefly, target areas were macro-dissected from unstained FFPE slides. After filter-aided sample preparation and desalting procedures, a liquid chromatography-tandem mass spectrometry (LC–MS/MS)-based proteomic study was conducted, using a Q Exactive HF-X Hybrid Quadrupole-Orbitrap mass spectrometer (Thermo Fisher Scientific, Waltham, MA) and an Ultimate 3000 RSLC system (Dionex, Sunnyvale, CA), according to the instructions of the manufacturer. The MaxQuant.Live version 1.2 (Max Planck Institute of Biochemistry, Munich, Germany) was used to perform BoxCar acquisition (19). The MS1 resolution was set to 120,000 at m/z 200 for BoxCar, and the acquisition cycle comprised two BoxCar scans at 12 boxes (scaled width, 1 Th overlap) with a maximum ion injection time of 20.8 per box with the individual AGC target set to 250,000. The MaxQuant version 1.6.1.0 (RRID : SCR_014485) (20) was employed with the Andromeda engine (21) to process the MS raw files. In the global parameter, the BoxCar was set as the experimental type. All search parameters were set as the default parameter of the software. For label-free quantification, the iBAQ algorithm was used as part of the MaxQuant platform (22). Raw LC–MS/MS data were uploaded into the PRIDE database (RRID : SCR_003411; Accession ID: PXD027602).

### Bioinformatic Analysis of the Proteomic Data

Proteomic data were analyzed using the Perseus software (RRID : SCR_015753, Max Planck Institute of Biochemistry). For comparisons, we performed an analysis of variance (ANOVA) and a two-sided t-test with a permutation-based false discovery rate (FDR) at significance level <0.05. Gene Ontology-biologic process (GOBP) and Gene Ontology-molecular function (GOMF) annotations were explicated using the Toppgene Suite (RRID : SCR_005726) (23). Protein–protein interaction (PPI) network models were constructed from the String database (24) and were illustrated using Cytoscape (RRID : SCR_003032) (25). The canonical pathway data were analyzed through the use of Ingenuity Pathway Analysis (Qiagen, RRID : SCR_008653, Hilden, Germany) (26).

### Clinical Validation of Risk Prediction Biomarkers of Inverted Urothelial Papilloma Using the TCGA Database

The TCGA bladder cancer (BLCA) dataset was used to determine the impact of the IUP-risk biomarkers on the prognosis of bladder cancer (27), under the R environment (R Foundation for Statistical Computing, RRID : SCR_001905, Vienna, Austria; packages “survival” and “survminer”). The RNA sequencing data were chosen for clinical validation because there is no publicly available cohort that contains both high-throughput proteomics and prognostic information in bladder neoplasms. For 405 patients, the gene expression and survival data were obtained from the cBioPortal for Cancer Genomics (https://docs.cbioportal.org/; RRID : SCR_014555) (28, 29). The prognostic effects of the log2-transformed gene expression levels were assessed by calculating a hazard ratio (HR) with 95% confidence interval (CI) using univariate Cox proportional hazard models. Kaplan–Meier analysis and log-rank test were used to compare overall survival outcomes, according to the low vs. high gene expression levels, using the median as a cutoff.

### Machine Learning-Based Stepwise Selection of Diagnostic Biomarkers for Inverted Urothelial Papilloma

First, we used a feature selection method for supporting vector machines with radial basis function kernel to choose the proteome with discriminative power between IUP and PUC (30). Next, the high-ranked proteins, selected from the machine learning analysis, were screened using The Human Protein Atlas (https://www.proteinatlas.org/; RRID : SCR_006710) (31, 32). Briefly, the antibody staining intensities (high, score 4; medium, score 3; low, score 2; not detected, score 1) were multiplied by the positive samples proportion showing each staining and then the scores were summed. For multiple antibodies, these scores were averaged. The finalized immunoscores in bladder urothelial carcinoma were compared against the relative fold changes derived from the t-test between IUP and PUC; when a protein was relatively overexpressed in urothelial carcinoma according to the public database but downregulated in PUC compared to IUP in the proteomic analysis, the protein was excluded. The finally selected markers were validated using immunohistochemistry.

### Immunohistochemical Validation of Diagnostic Biomarker of Inverted Urothelial Papilloma

A validation cohort, consisting of IUP (n = 25) and PUC with inverted growth (n = 16), was utilized independently from those used for the proteomic analysis. Immunostaining assays for SERPINH1 (1:200, sc-5293, RRID : AB_627757, Santa Cruz Biotechnology, Dallas, TX) and PYGB (1:2,000, HPA031067, RRID : AB_2673722, Sigma-Aldrich, St. Louis, MO) were conducted in IUP and PUC with inverted growth, using an automated BenchMark ULTRA System (Roche Diagnostics, Rotkreuz, Switzerland). The immunostained glass slides were digitally scanned using an Aperio Digital Pathology Slide Scanner AT2 (Leica Biosystems, Buffalo Grove, IL). Expression of SERPINH1 and PYGB was quantified by “H-score” [1 ∗ (% cells 1+) + 2 ∗ (% cells 2+) + 3 ∗ (% cells 3+)], with an interpretation ranging from 0 to 300 (33), using the QuPath platform for bioimage analysis (RRID : SCR_018257) (34). The area under the receiver operating characteristic (AUROC) was calculated using MedCalc version 20.019 (MedCalc Software Ltd, RRID : SCR_015044, Ostend, Belgium) and the optimal level of H-score with corresponding sensitivity and specificity were estimated on the basis of the Youden index.

## Results

### Proteomic Signatures Divide Low Risk and High Risk in Inverted Urothelial Papilloma

**Figure 1:**
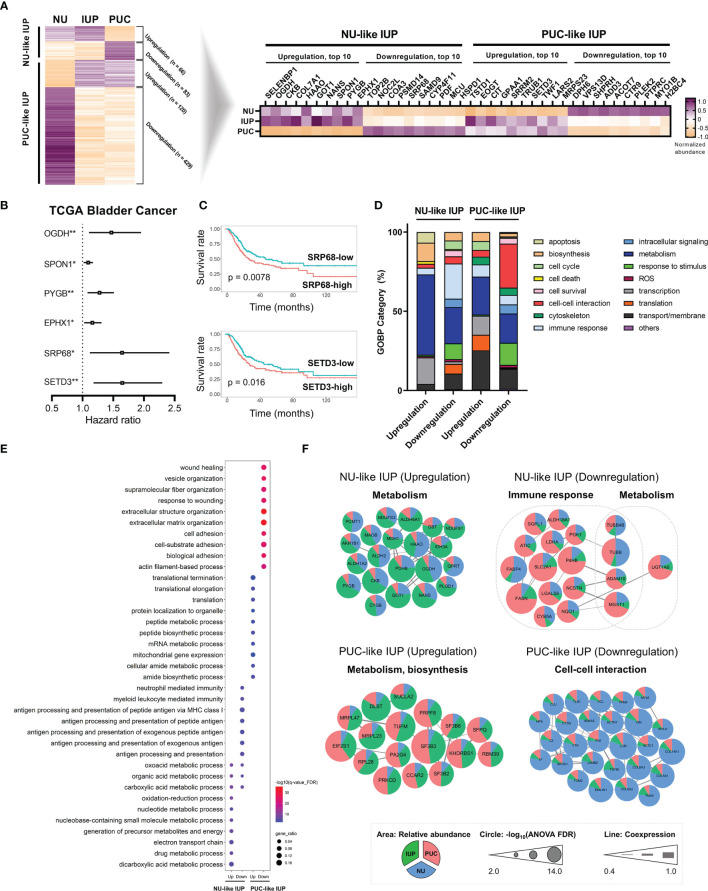
Proteomics-based oncologic signatures of inverted urothelial papilloma (IUP). **(A)** ‘Papillary urothelial carcinoma (PUC)-like’ IUP and ‘normal urothelium (NU)-like’ IUP signatures selected from the ANOVA-based differentially expressed proteins (left). The 40 top-ranked proteins of each signature are summarized (right). **(B)** Hazard ratios with 95% confidence intervals of the significant univariate Cox analysis results of the 40 proteins in the TCGA bladder cancer (BLCA) dataset. (*p < 0.05, **p < 0.01). **(C)** Kaplan–Meier graphs of SRP68 and SETD3 in the TCGA BLCA dataset. **(D)** Distribution of Gene Ontology-Biologic Process (GOBP) categories enriched in the upregulated and downregulated proteomes of ‘NU-like’ IUP and ‘PUC-like’ IUP signatures. **(E)** The top 10 significantly associated GOBPs. **(F)** Protein–protein interaction networks of the proteomes included in the top 10 significant GOBPs and their related functions. Unconnected proteins are not presented.

Overall, the LC–MS/MS proteomic assay identified 9,890 and quantified 5,057 proteins, which were present in ≥20% of all samples, from peptides with high confidence (FDR <0.01). The normalized protein abundance is provided as **Supplementary Table S2**. Principal component analysis demonstrated that IUP was closer to PUC than NU (**Supplementary Figure S2**). Using a one-way ANOVA test among the three groups (**Supplementary Figure S3A**), 698 differentially expressed proteins (DEPs) were identified, and these proteins were used to stratify the risks in IUP compared with PUC and NU (**Figure 1A**, left). Specifically, ‘NU-like’ IUP signatures, namely, upregulation of SELENBP1, OGDH, and CKB (total, n = 66) and downregulation of TOP2B, NOC2L, and COA3 (total, n = 83), were similar between IUP and NU, as opposed to PUC (**Figure 1A**, left). On the other hand, ‘PUC-like’ IUP signatures, namely, upregulation of TSTD1, EOGT, and CIT (total, n = 120) and downregulation of DPH6, VPS13D, and SHPRH (total, n = 429), were similar between IUP and PUC, as opposed to NU (**Figure 1A**, left). We investigated the clinical significance of the 40 most significant proteins of the ‘NU-like’ and ‘PUC-like’ IUP signatures (**Figure 1A**, right) through survival analysis using the TCGA database. Univariate Cox analysis identified the significance impact of OGDH (HR = 1.469, 95% CI = 1.105–1.954, p = 0.008), SPON1 (HR = 1.092, 95% CI = 1.019–1.170, p = 0.012), PYGB (HR = 1.277, 95% CI = 1.080–1.511, p = 0.004), EPHX1 (HR = 1.159, 95% CI = 1.026–1.309, p = 0.017), SRP68 (HR = 1.643, 95% CI = 1.118–2.415, p = 0.011), and SETD3 (HR = 1.647, 95% CI = 1.180–2.299, p = 0.003) expression on poor urothelial carcinoma outcomes (**Figure 1B**). Among these proteins, low expression of SRP68 and high expression of SETD3 were concordantly observed in the ‘NU-like’ and ‘PUC-like’ IUP signatures, respectively (**Figure 1A**, right). These results are consistent with the ones derived from the BCLA survival analysis using the TCGA data; in the latter, low SRP68 expression was associated with favorable, whereas high SETD3 with poor prognosis (**Figure 1C**).

We investigated the molecular functions associated with upregulated and downregulated proteomes in each group. Overall, the GOBP analysis showed significant enrichment of metabolism in all signatures and especially in the upregulated ‘NU-like’ IUP signatures (**Figure 1D**). The downregulated proteins of the ‘NU-like’ IUP signatures were also enriched in immune response, the upregulated ‘PUC-like’ IUP signatures were biased towards transport/membrane, transcription, and translation, while the downregulated ‘PUC-like’ IUP signatures were enriched in cell–cell interaction, responses to stimuli, and transport/membrane functions (**Figure 1D**). **Figure 1E** summarizes the top 10 significant GOBP terms of each signature set that showed concordant membership. The PPI networks of the proteins included in these top 10 GOBPs consistently highlighted metabolism in the upregulated ‘NU-like’ IUP proteins, immune response and metabolism in the downregulated ‘NU-like’ IUP proteins, metabolism and biosynthesis in the upregulated ‘PUC-like’ IUP proteins, and cell–cell interaction in the downregulated ‘PUC-like’ IUP proteins (**Figure 1F**). Previous studies identified metabolism, cell proliferation, immune response, and intercellular communication as constitutively altered functions in urothelial carcinoma (35, 36). Enhanced metabolism/biosynthesis functions and decreased cell–cell interaction/adhesion, consistent with what was found in the ‘PUC-like’ IUP signatures, were previously shown to promote PUC by supporting cell proliferation and structural breakdown (35, 37–39). Therefore, the results suggested altered metabolism, biosynthesis, and cell–cell interaction functions were consistent with the ‘PUC-like’ (high-risk) IUP.

### Activation of Metabolism and Inhibition of Structure-Related Processes Are Distinctive Functions of Inverted Urothelial Papilloma

**Figure 2:**
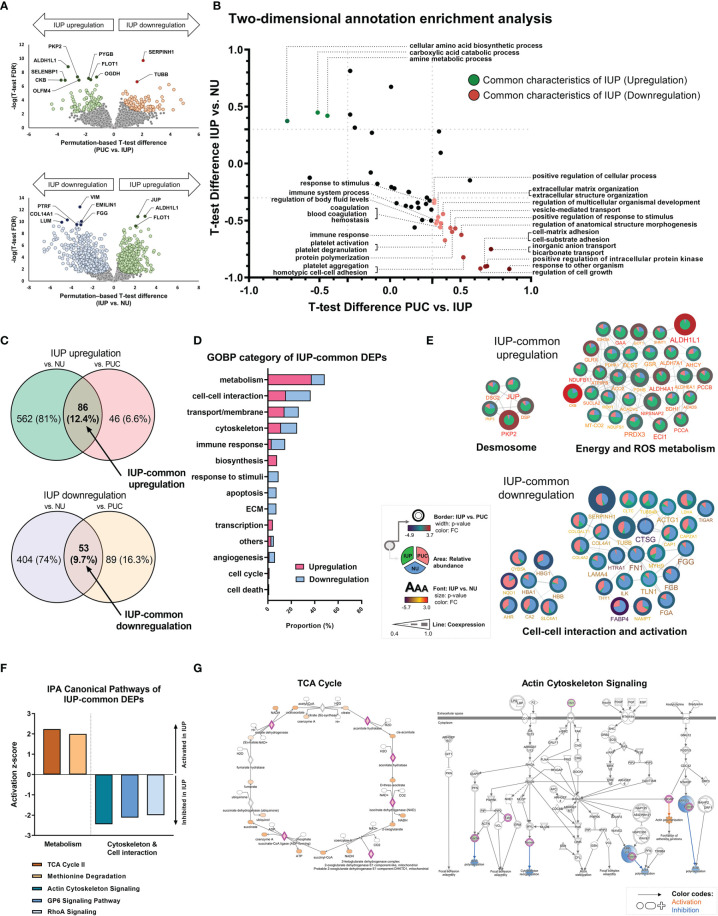
Unique functional profiles of inverted urothelial papilloma (IUP). **(A)** Differentially expressed proteins (DEPs) identified by using t-tests between IUP and papillary urothelial carcinoma (PUC) (upper) and between IUP and normal urothelium (NU) (lower). **(B)** Matched Gene Ontology-Biologic Processes (GOBPs) commonly enriched in the DEPs from both comparisons (PUC vs. IUP and IUP vs. NU). **(C)** DEPs commonly upregulated or downregulated in IUP compared with NU and PUC (IUP-common DEPs). **(D)** GOBP categories related to the IUP-common DEPs. **(E)** Protein–protein interaction networks of the upregulated and downregulated IUP-common DEPs and their related functions. Unconnected proteins are not presented. **(F)** Ingenuity Pathway Analysis (IPA)-canonical pathways predicted to be activated (metabolism) or inhibited (cytoskeleton and cell–cell interaction) in IUP. **(G)** Detailed IPA pathways (TCA and actin-cytoskeleton signaling) showing activated (orange) or inhibited (blue) components in IUP.

To further characterize the distinct pathobiology of IUP, we identified DEPs (permutation-based t-test FDR <0.05) between IUP and PUC, namely, PKP2, PYGB, SERPINH1, and TUBB, and those between IUP and NU, namely, ALDH1L1, JUP, COL14A1, and VIM (**Figure 2A** and **Supplementary Figures S3A, B**). GOBP-based 2D annotation enrichment analysis, as previously described (40), revealed that IUP was distinctly enhanced in the metabolism of amines and carboxylic acids yet repressed in cell response, transport/membrane, adhesion, interaction, and extracellular matrix (ECM) (**Figure 2B**). Similarly, IUP-common DEPs, or the intersecting proteins derived from the comparisons of IUP with PUC or NU, jointly coded for similar GOBP themes relevant to the oncologic significance of IUP as mentioned earlier, namely, metabolism, cell–cell interaction, cytoskeleton formation, and transport/membrane (**Figures 2C, D** **)**. The representative IUP-common DEPs selected from the top 10 most significant GOBPs, namely, PKP2, ALDH1L1, CKB, SERPINH1, and TUBB, interacted towards upregulated processes related to desmosome formation or metabolism and downregulated processes related to cell–cell interaction, cell activation, and ECM (**Figure 2E**). In line with this, IPA for these IUP-common DEPs confirmed the activation of metabolism (z-score ≥2.0) and inhibition of cytoskeleton formation/cell–cell interaction (z-score ≤−2.0) as the constitutive pathways in IUP (**Figure 2F**). **Figure 2G** illustrates the activation of the TCA cycle and inhibition of the actin-cytoskeleton signaling in the IUP-common DEPs.

### Proteome-Based Machine Learning Analysis Identified Candidate Biomarkers for the Diagnosis of Inverted Urothelial Papilloma

**Figure 3:**
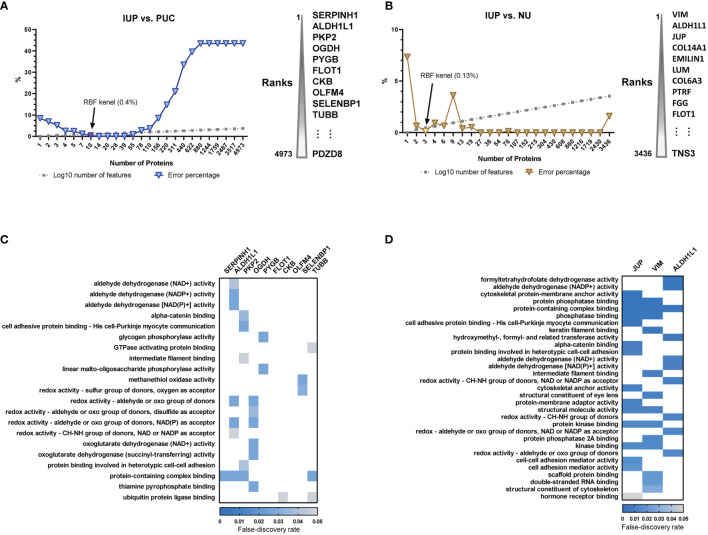
Biomarker discovery of inverted urothelial papilloma (IUP) diagnosis. **(A)** The 10 optimal biomarkers of IUP vs. papillary urothelial carcinoma (PUC), identified by a support vector machine. **(B)** The three optimal biomarkers of IUP vs. normal urothelium (NU) identified by a support vector machine. **(C)** Gene Ontology-Molecular Functions (GOMFs) enriched in the 10 optimal biomarkers of IUP vs. PUC. **(D)** GOMFs enriched in the three optimal biomarkers of IUP vs. NU.

To translate the findings from proteomics to IUP diagnosis in practice, we selected proteome features discriminating IUP from PUC and NU, using support vector machine-based machine learning. The lowest error rates, along with keeping the protein lists short, were achieved at 0.4% between IUP and PUC, corresponding to 10 proteins (**Figure 3A**), and at 0.13% between IUP and NU, corresponding to 3 proteins (**Figure 3B**). We failed to find GOBPs implicated in these proteome sets due to the small numbers. However, GOMF analysis identified aldehyde, glycogen, and redox metabolism enriched for the proteomes of IUP compared to PUC (**Figure 3C**), whereas aldehyde metabolism, cytoskeleton, and cell–cell interaction overrepresented by the proteomes of IUP compared to NU (**Figure 3D**). The results corroborated the indispensable roles of metabolism and cytoskeleton/cell interaction-related functions in IUP compared to PUC and NU.

### PYGB Distinguishes Inverted Papilloma From Inverted Papillary Urothelial Carcinoma

**Figure 4:**
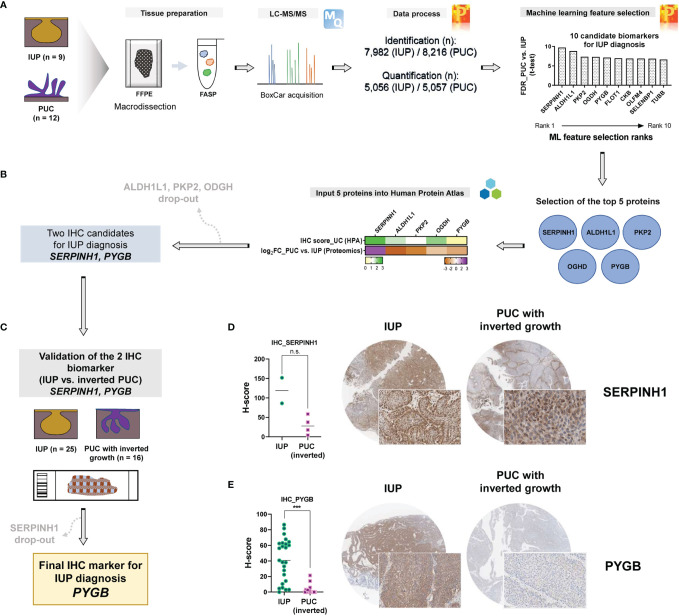
Identification of PYGB as an accurate biomarker to differentiate inverted urothelial papilloma (IUP) from papillary urothelial carcinoma (PUC) with inverted growth. **(A)** Summary of the machine learning feature selection of 10 candidate biomarkers. **(B)** Selection of the five top-ranked proteins and further narrowing-down to the two proteins (SERPINH1 and PYGB), based on The Human Protein Atlas. **(C)** Immunohistochemical validation of SERPINH1 and PYGB in an independent cohort of IUP and PUC with inverted growth. **(D)** SERPINH1 immunostaining in IUP vs. PUC with inverted growth (Mann–Whitney p = 0.1333; n.s., not significant). **(E)** PYGB immunostaining in IUP vs. PUC with inverted growth (***Mann–Whitney p < 0.0001).

To prioritize protein biomarkers for IUP diagnosis, the top 10 candidates selected by machine learning analysis between IUP and PUC were further shortlisted, based on the t-test FDR and the machine learning rank order (**Figure 4A**), resulting in the five most robust proteins; SERPINH1, ALDH1L1, PKP2, OGDH, and PYGB (**Figure 4B**). These were additionally narrowed down, based on the similarity between the proteomic profiles and knowledge-based immuno-expression in bladder urothelial carcinoma (**Figure 4B**, green heatmap); ALDH1L1, PKP2, and OGDH were excluded due to the discordancy. Finally, SERPINH1 and PYGB were selected as candidate biomarkers for the distinction between IUP and PUC (**Figure 4B**).

Differential diagnosis of IUP and PUC with inverted growth is one of the common pitfalls in pathological diagnosis of bladder neoplasms due to the morphologic similarity (3, 11, 12). We aimed to resolve this challenge by validating SERPINH1 and PYGB using immunohistochemistry in IUPs and PUCs with inverted growth. To achieve our goal, we additionally enrolled an independent cohort comprising IUP (n = 25) and PUC with inverted growth (n = 16) (**Figure 4C**). In a pilot test, discordantly to the proteomic analysis, SERPINH1 appeared to be diffusely expressed in IUPs compared to PUCs with inverted growth (Mann–Whitney p = 0.1333; **Figure 4D**); SERPINH1 was precluded from further study. However, PYGB was significantly upregulated in IUP compared with PUC with inverted growth (Mann–Whitney p <0.0001; **Figure 4E**), verifying the differential expression found in the proteomic data. The AUROC for the diagnosis of IUP vs. PUC with inverted growth using the PYGB H-score was 0.923 (p <0.0001) and the sensitivity and specificity were 72% (95% CI = 50.6–87.9%) and 100% (95% CI = 79.4–100%), respectively, when H-score 21.4 was set as the cutoff (**Supplementary Figure S4**). The morphology and clinical follow-up of IUPs were similar regardless of whether the immunostaining to PYGB was positive or not. Therefore, we propose that PYGB might be a useful immunohistochemical biomarker for differentiation of IUP from low-grade PUC with inverted growth.

## Discussion

In the current study, we applied in-depth proteomics-based machine learning analysis and presented two novel findings as follows: 1) the comprehensive proteomic landscape of IUP to stratify its oncologic risk by identifying two subgroups, a low-risk and a high-risk and 2) a novel immunohistochemical biomarker PYGB to differentiate IUP from PUC with inverted growth.

The oncologic risk of IUP has been controversial in the literature (41–44). Up to 10% of IUPs have been reported to eventually progress into urothelial carcinoma (3). Our in-depth proteomic analysis indicated the presence of a high-risk IUP subgroup, sharing similar proteomic landscapes with PUC. First, based on the similarity of the 698 DEPs found among the three groups, the proteomic profile of IUP was clearly clustered into two risk-stratifying subgroups; the ‘NU-like’ IUP signatures that were most likely to indicate a low-risk group and the ‘PUC-like’ IUP signatures that were suspected to indicate an aggressive tumor behavior. The ‘NU-like’ IUP signatures included upregulation of proteins related to tumor-suppressive functions, namely, SELENBP1, OGDH, CKB, and GOT1 (45–48), besides downregulation of proteins related to oncogenic property, such as TOP2B, NOC2L, PSMD14, SRP68, CYP4F11, PDF, MCU, and HSPD1 (48–54). On the contrary, the ‘PUC-like’ IUP signatures prioritized proteins previously implicated in cancer promotion, including enrichment of oncogenes [CIT, GPAA1, SRRM2, SETD3, TWF1, and MRPS23 (55–60)] and low expression of tumor-suppressor proteins [SHPRH and ADD3 (61, 62)]. Especially, the survival analysis of the BLCA TCGA dataset further validated SRP68 and SETD3 as potential predictive candidates for the ‘NU-like’ (low-risk) or ‘PUC-like’ (high-risk) IUP. SRP68 is a key component of SRP ribonucleoprotein complex regulating endoplasmic reticulum trafficking for protein export and tumor cell mobility (63–65). We showed that low *SRP68* mRNA expression was significantly associated with favorable BLCA prognosis in the TCGA dataset. A previous study also demonstrated that SRP68 was upregulated in bladder cancer compared with adjacent normal tissue and also suggested a key oncogenic function of SRP68 in urothelial carcinoma; this is concordant with our study, where SRP68 was highly expressed in PUC compared to the other two groups (51). High abundance of SETD3, one of the top proteins for high-risk ‘PUC-like’ IUP signatures, was associated with poor prognosis in the TCGA BLCA dataset. As an epigenetic regulator, SETD3 was previously suggested as a crucial oncogenic modulator in bladder cancer (56). Taken together, the application of SRP68 and SETD3 might be considered as potential biomarkers for identifying the high-risk IUPs.

In this study, a Gene Ontology analysis presented the biologic networks of the ‘PUC-like’ IUP signatures; these were characterized by alteration of metabolism, biosynthesis, and cell–cell interaction. Concordant findings have been reported in previous studies, showing alteration of macromolecules/metabolites and structural frameworks associated with tumorigenesis and progression of PUC (35, 37). In addition, membrane and transport functions were also enriched in the ‘PUC-like’ IUP signatures, also in accordance with a prior study that showed alteration in membrane transporters was associated with the malignant behavior of urothelial carcinoma (66). With the enrichment analysis, therefore, we suggest that unique cellular processes might promote an aggressive behavior of IUPs.

Moreover, using commonly expressed proteins in IUP based on the cross-comparison of the DEPs between IUP and NU or IUP and PUC, we also identified unique biological characteristics of IUP. Interestingly, the aforementioned GOBP functions related to the oncologic signatures of IUP, namely, metabolism, cell–cell interaction, and transport/membrane, were similarly enriched here. These results further support the innate significance of these functions in the tumor biology and the potential transformation of IUP. Also, the altered themes of transport/membrane, immune response, and response to stimuli in the IUP-common DEPs may reflect the hyperplastic process in reaction to inflammation, infection, and environmental stress previously suggested regulating IUP pathogenesis (3).

For the first time, along with proteome-based biologic analysis, we successfully discovered PYGB as a novel specific biomarker for the differentiation of IUP from low-grade PUC with inverted growth, using a machine learning feature selection and immunohistochemical validation. PYGB is a brain form of glycogen phosphorylase that supports survival and proliferation of various cell types (67). Glycogen comprises the major glucose storage, and glycogen metabolism balances glucose utilization and energy production (68). Glycogen phosphorylase regulates debranching of glycogen, mobilizing glucose to enter glycolysis pathway or a pentose-phosphate shunt (67). Recent studies revealed that IUP harbors frequent *HRAS* mutation at higher rates than that observed in PUC with or without inverted growth (6, 11). While uncontrolled activation of the RAS pathway transduces cellular proliferation in IUP (69), upregulated PYGB presumably fuels energy and anabolic sources required for the growth of IUP and downregulation of PYGB in PUC might concur with the metabolic reprogramming (67). In addition, altered energy and ROS metabolism, enriched in the upregulated IUP-common proteome, might corroborate the lack of glycogen deposit in IUP associated with hypoxia (68).

There are limitations in the study. Due to the rarity, the number of IUP included in the validation of the immunostaining for PYGB is relatively small. We are planning an external validation study to confirm the usefulness of PYGB by involving multiple institutions. In addition, the functional role of PYGB in IUP was not validated because *in vitro* or *in vivo* models of IUP are not available. Further studies are needed to confirm the biofunctions of PYGB in IUP.

## Conclusion

In conclusion, we comprehensively investigated the in-depth proteomic landscape of IUP and found proteome signatures associated with oncologic risk. For the first time, we also discovered PYGB as an accurate biomarker to differentiate IUP from low-grade PUC with inverted growth. In difficult cases, a novel immunohistochemical biomarker such as PYGB can be particularly helpful to establish an accurate diagnosis and prevent a potentially unnecessary treatment.

## Data Availability Statement

The datasets presented in this study can be found in online repositories. Proteomic data are available via ProteomeXchange (http://www.proteomexchange.org/) with identifier PXD027602.

## Ethics Statement

The studies involving human participants were reviewed and approved by the Institutional Review Board, Seoul National University Hospital. Written informed consent for participation was not required for this study in accordance with the national legislation and the institutional requirements.

## Author Contributions

MJ and HSR designed the research, analyzed the data, and drafted the paper. CL, DH, KK, SY, KCM, HK, MJS, and BK were responsible for data collection and analysis. DH, KK, and SY were responsible for statistical analysis. MJ, IPN, HL, and HSR contributed to the study design and revised the manuscript. All authors listed have made a substantial, direct, and intellectual contribution to the work and approved it for publication.

## Funding

This research was funded by the National Research Foundation of Korea (NRF) funded by the Ministry of Science, ICT and Future Planning (NRF-2019R1C1C1006640, 2021R1F1A1063982, 2021R1A2C4086635, and 2022R1A2C4001439).

## Conflict of Interest

The authors declare that the research was conducted in the absence of any commercial or financial relationships that could be construed as a potential conflict of interest.

## Publisher’s Note

All claims expressed in this article are solely those of the authors and do not necessarily represent those of their affiliated organizations, or those of the publisher, the editors and the reviewers. Any product that may be evaluated in this article, or claim that may be made by its manufacturer, is not guaranteed or endorsed by the publisher.
