# Supplementary material for: Proteomic-Based Machine Learning Analysis Reveals PYGB as a Novel Immunohistochemical Biomarker to Distinguish Inverted Urothelial Papilloma From Low-Grade Papillary Urothelial Carcinoma With Inverted Growth
Source: Front Oncol. 2022 Mar 24;12:841398. doi: 10.3389/fonc.2022.841398 (PMC8987228; doi:10.3389/fonc.2022.841398)
Supplement: Supplementary file 1 [file DataSheet_1.docx]

Supplementary Material

# Supplementary Table S1. Demographic and pathological details

| **Variables** | **Proteomic analysis (n = 31)** | | | **Immunohistochemical validation (n = 41)** | |
| --- | --- | --- | --- | --- | --- |
|  | **Inverted urothelial papilloma** | **Papillary urothelial carcinoma** | **Normal urothelium** | **Inverted urothelial papilloma** | **Papillary urothelial carcinoma with inverted growth** |
| Number | 9 | 12 | 10 | 25 | 16 |
| Sex (male, %) | 100% | 91.7% | 50% | 92% | 75% |
| Age (median, range) | 56 (42-68) | 63.5 (39-77) | 64 (36-76) | 52 (24-86) | 62.5 (31-74) |
| T stage | n.a. | Ta (100%) | n.a. | n.a. | Ta (100%) |
| WHO grade | n.a. | Low (16.7%)  High (83.3%) | n.a. | n.a. | Low (81.2%)  High (18.8%) |

n.a: not applicable

# Supplementary Figure

**Supplementary Figure S1.** Representative photomicroscopy of inverted urothelial papillomas in each column. (original magnification: x40, top and x200, bottom)

**Supplementary Figure S2.** Principal component analysis of the proteomic profiles of inverted urothelial papilloma (IUP), papillary urothelial carcinoma (PUC), and normal urothelial (NU)

**Supplementary Figure S3.** **(A)** An ANOVA test of inverted urothelial papilloma (IUP), normal urothelium (NU), and papillary urothelial carcinoma (PUC). **(B)** Differentially expressed proteins (DEPs) from a t-test between IUP and NU. **(C)** DEPs from a t-test between IUP and PUC.

**Supplementary Figure S4.** ROC curve for the diagnosis of inverted urothelial papilloma vs. papillary urothelial carcinoma with inverted growth using the PYGB H-scores.
